# Comparative Genome analysis of the Genus *Curvibacter* and the Description of *Curvibacter microcysteis* sp. nov. and *Curvibacter cyanobacteriorum* sp. nov., Isolated from Fresh Water during the Cyanobacterial Bloom Period

**DOI:** 10.4014/jmb.2306.06017

**Published:** 2023-08-21

**Authors:** Ve Van Le, So-Ra Ko, Mingyeong Kang, Seonah Jeong, Hee-Mock Oh, Chi-Yong Ahn

**Affiliations:** 1 Cell Factory Research Centre, Korea Research Institute of Bioscience and Biotechnology, Daejeon 34141, Republic of Korea; 2 Department of Environmental Biotechnology, KRIBB School of Biotechnology, University of Science and Technology, Daejeon 34113, Republic of Korea

**Keywords:** *Curvibacter*, cyanobacterial bloom, *Microcystis*

## Abstract

The three Gram-negative, catalase- and oxidase-positive bacterial strains RS43^T^, HBC28, and HBC61^T^, were isolated from fresh water and subjected to a polyphasic study. Comparison of 16S rRNA gene sequence initially indicated that strains RS43^T^, HBC28, and HBC61^T^ were closely related to species of genus *Curvibacter* and shared the highest sequence similarity of 98.14%, 98.21%, and 98.76%, respectively, with *Curvibacter gracilis* 7-1^T^. Phylogenetic analysis based on genome sequences placed all strains within the genus *Curvibacter*. The average nucleotide identity (ANI) and digital DNA–DNA hybridization (dDDH) values between the three strains and related type strains supported their recognition as two novel genospecies in the genus *Curvibacter*. Comparative genomic analysis revealed that the genus possessed an open pangenome. Based on KEGG BlastKOALA analyses, *Curvibacter* species have the potential to metabolize benzoate, phenylacetate, catechol, and salicylate, indicating their potential use in the elimination of these compounds from the water systems. The results of polyphasic characterization indicated that strain RS43^T^ and HBC61^T^ represent two novel species, for which the name *Curvibacter microcysteis* sp. nov. (type strain RS43^T^ =KCTC 92793^T^=LMG 32714^T^) and *Curvibacter cyanobacteriorum* sp. nov. (type strain HBC61^T^ =KCTC 92794^T^ =LMG 32713^T^) are proposed.

## Introduction

The genus *Curvibacter*, affiliated to the family *Comamonadaceae* of the phylum *Proteobacteria*, was originally described in 2004, with *Curvibacter gracilis* as the type species [1]. Since then, four species with validly published names [2] have been isolated from well water [1,3] and distilled water [4]. Species of the genus *Curvibacter* described to date are Gram-negative, curve rod-shaped, and positive for catalase and oxidase. The members of the genus *Curvibacter* commonly contain C_16:0_, C_16:1_, C_18:1_, and C_8:0_ 3-OH as their major fatty acids and Q-8 as their predominant respiratory quinone [1]. Currently, no comparative genome analysis has been carried out on the genus *Curvibacter*.

Cyanobacteria play an essential role in the carbon and nutrient cycles [5]. However, they cause harm to ecosystems and humans through the production of cyanotoxins [6]. The excessive proliferation of cyanobacteria, also known as cyanobacterial bloom, destroys biodiversity and degrades drinking water quality [6]. During the study of the role of bacterial community in the formation of cyanobacterial blooms, three new strains, RS43^T^, HBC28, and HBC61^T^, were isolated from a freshwater reservoir during the cyanobacterial bloom period. The present study aimed to determine their taxonomic status using polyphasic approach. In addition, the first comparative genomic study for the genus *Curvibacter* was performed to predict their ecological functions and potential biotechnological applications.

## Materials and Methods

### Isolation and Cultivation

Strains RS43^T^, HBC28, and HBC61^T^ were isolated from Daechung Reservoir (36° 22' 33.7'' N, 127° 38' 20.6'' E) according to the procedures previously described [7]. Briefly, the sample was diluted by the standard ten-fold serial dilution method and was spread on agar plates of Reasoner’s 2A (R2A). After incubation at 25°C for 1 week under aerobic conditions, colonies were picked and purified two times by repeated subculture. The strains were then preserved in R2A medium containing 20% glycerol (v/v) at –80°C. Considering the close phylogenetic relationships, *Curvibacter gracilis* KCTC42831^T^ (=7-1^T^), *Curvibacter lanceolatus* KCTC42829^T^ (=IAM 14947^T^), and *Curvibacter delicatus* KCTC42828^T^ (=IAM 14955^T^) procured from KCTC were chosen as reference strains and they were compared in the aspect of polyphasic characterization.

### Molecular Identification, Genome Sequencing, and Pangenome Analysis

The whole genomic DNA was extracted using FastDNA Spin Kit for Soil (MB Biomedicals, USA). For strain identification, the 16S rRNA gene was amplified and sequenced with the universal bacterial primers of 27F, 518F, 785F, 805R, 907R, and 1492R [8-10]. Pairwise comparisons of the almost-complete 16S rRNA gene sequences of three strains and those of related taxa were performed on the latest updated EzBioCloud (www.ezbiocloud.net)[11] and NCBI blast (www.ncbi.nlm.nih.gov). The draft genomes of three strains were sequenced using the Illumina MiSeq (Macrogen Inc., South Korea) platform and annotated by Prokka v.1.12 and Rapid Annotation using Subsystems Technology (RAST). The metabolic pathways of strains RS43^T^, HBC28, and HBC61^T^ were reconstructed using BlastKOALA [12]. The G+C content was calculated from the whole genome sequence. For comparative genome analysis, the genomes of *Curvibacter* species were obtained from NCBI (Table 1). Pangenomic analyses were performed using the EDGAR 3.0 platform [13]. The biosynthetic gene clusters of secondary metabolites were searched using AntiSMASH 7 beta [14] with strict detection.

### Phylogeny

The 16S rRNA gene sequences of three strains and other closely related strains obtained from the GenBank/EMBL/DDBJ databases were aligned using the MUSCLE algorithm. To identify the phylogenetic location of three strains, phylogenetic trees were constructed by using maximum-likelihood [15], neighbor-joining [16], and minimum-evolution [17] methods with the software MEGA version 11 [18]. The confidence levels and distance matrices of phylogenetic trees were determined with 1000 bootstrap replicates and Kimura’s two-parameter model [19], respectively. To provide cogent evidence for the assignment of strains RS43^T^, HBC28, and HBC61^T^ as novel species, the ANI and dDDH values were calculated using the OrthoANI algorithm and the Genome-to-Genome Distance Calculator (GGDC 2.0) [20], respectively. The two-way average amino acid identity (AAI) was calculated using AAI calculator (http://enve-omics.ce.gatech.edu/aai/) and percentage of conserved proteins (POCP) values were by using EDGAR 3.0 platform [13]. A phylogenomic tree was built utilizing the Type (Strain) Genome Server (TYGS) [21].

### Phenotypic and Biochemical Properties

Morphological characteristics of strains RS43^T^, HBC28, and HBC61^T^ were visualized under a CM-20 transmission electron microscope (Philips) after 3-day incubation at 25°C on R2A medium. Cell motility was identified using the hanging-drop method [22]. A Gram stain kit (Becton Dickinson, USA) was used to determine the Gram reaction. Catalase and oxidase tests were done using 3% (v/v) H_2_O_2_ and 1% (w/v) N, N, N’, N’-tetramethyl-1,4-phenylenediamine, respectively. The growth of three strains on R2A agar, Luria–Bertani agar (LBA, Difco, USA), tryptone soy agar (TSA, Difco, USA), and nutrient agar (NA, Difco, USA) was investigated at 25°C for five days. Growth tolerance ranges for temperature, pH, and NaCl were assessed by observing colony growth on R2A agar plates after 5-day incubation. The growth temperature was evaluated at 4, 10, 17, 20, 25, 30, 37, 40, and 60°C. Growth at different pH (4.0–12.0 with an interval of 1.0) was assessed by adjusting the pH using the buffer system [7]. The tolerance range for NaCl was determined by culturing strains on R2A agar plates supplemented with varying amounts of NaCl [0.0, 0.1, 0.3, 0.5, 1.0, 1.5, and 2.0% (w/v)]. Biochemical characteristics and enzyme activities were inspected using API ZYM and API 20NE kits (bioMérieux, France). Hydrolysis of Tween 20 (1.0%, w/v), Tween 80 (1.0%, w/v), and skim milk (0.5%, w/v) was tested after 3-day incubation [23].

### Chemotaxonomic Characterization

For chemotaxonomic analysis, all strains were grown on R2A for 3 days at 25°C. After harvesting the cells, the fatty acids were saponified, methylated, extracted, and analyzed according to the protocol recommended by MIDI Microbial Identification System (version 6.1, MIDI) [24]. Polar lipids were extracted from lyophilized cells, separated and identified as described previously [25–28]. Quinones were extracted and purified with a mixture of chloroform and methanol (2:1) for 3–4 h and analyzed by HPLC [29].

## Results and Discussion

### Phylogenetic Characteristics

**Fig. 1:**
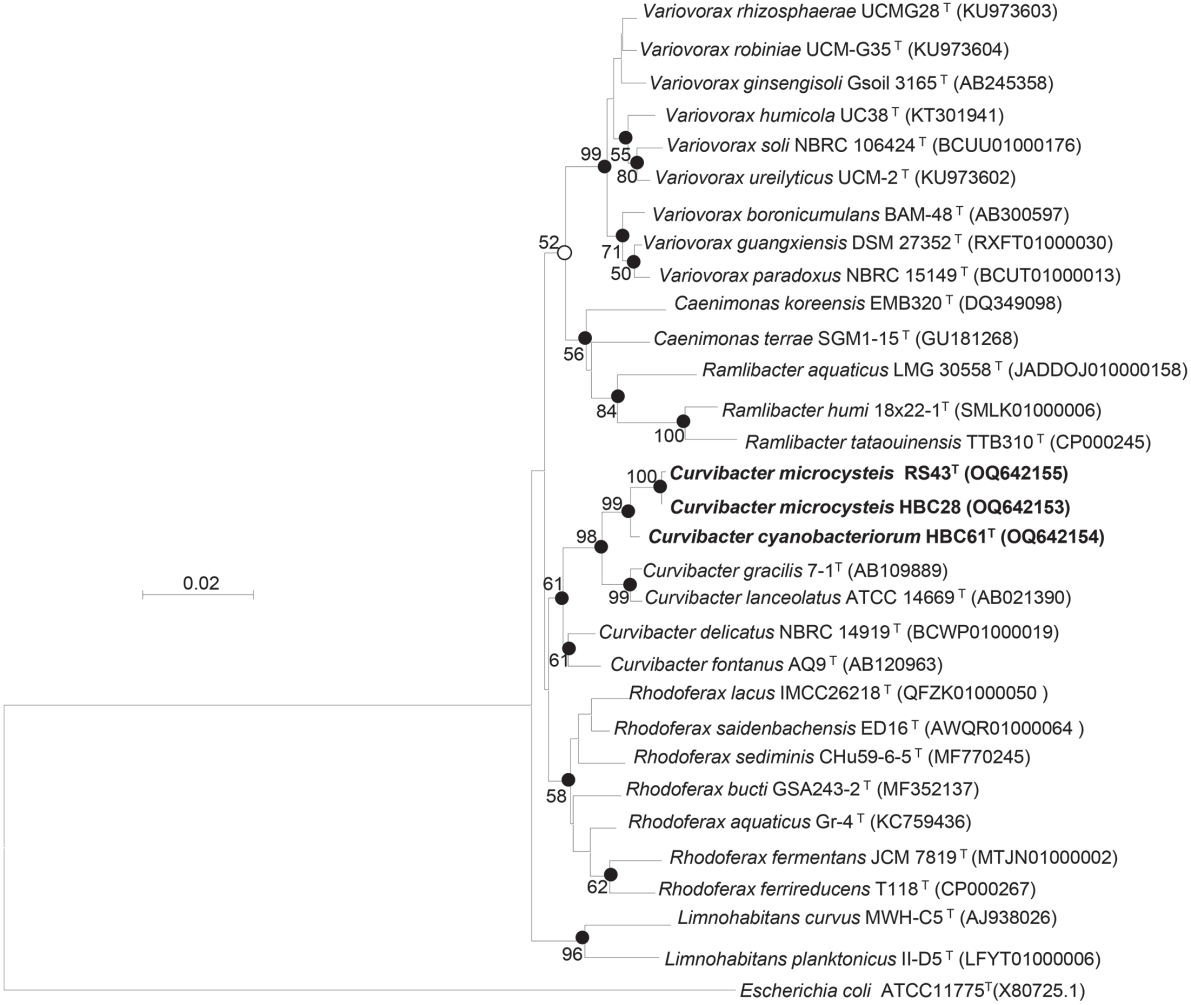
Neighbor-joining phylogenetic tree based on the 16S rRNA gene sequences depicting the phylogenetic placements of three strains among the related species. Bootstrap values above 50% are shown at branch points. Closed circles indicate that the corresponding nodes were also recovered in the maximum-likelihood and minimum-evolution methods, whereas the open circles indicate nodes recovered with neighbor-joining and maximum-likelihood algorithms.

The 16S rRNA gene sequences of strains RS43^T^, HBC28, and HBC61^T^ determined by the Sanger sequencing were 1474, 1484, and 1471 bp in length, respectively. Strains RS43^T^, HBC28, and HBC61^T^ were most closely related to *Curvibacter gracilis* 7-1^T^ with 16S rRNA sequence similarities of 98.14%, 98.21%, and 98.76%, respectively. The 16S rRNA gene sequence of strains RS43^T^ and HBC61^T^ shared 99.35% similarity with each other. The phylogenetic trees revealed that the three strains formed a coherent cluster with *C. gracilis* 7-1^T^ and *Curvibacter lanceolatus* IAM 14947^T^. This relationship was supported by high bootstrap percentages (Fig. 1). Therefore, the three strains were phylogenetically affiliated with the genus *Curvibacter*.

### Genomic Characteristics

The draft genome sequences of strains RS43^T^, HBC28, and HBC61^T^ had a total nucleotide length of 4,895,745 bp (24 contigs), 4,822,995 bp (21 contigs), and 4,848,818 bp (19 contigs), respectively, with the sequencing depth of 148×, 147×, and 147×, respectively. Overall, the draft genome met the criteria required for the taxonomic purposes proposed [30]. The G+C contents of strains RS43^T^ and HBC28 were estimated to be 65.22 % and 65.29 %, which correspond with the G+C content range (62.2–66.0 mol%) of members of the genus *Curvibacter* [1].

The genomic features of *Curvibacter* species are presented in Table 1. The draft genome sequences of *Curvibacter* species vary significantly in size, ranging from 3.78 Mbp for *Curvibacter delicatus* NBRC 14919^T^ to 6.83 Mbp for *Curvibacter lanceolatus* ATCC 14669^T^. Additionally, the DNA G+C contents varied among the strains, with *C. delicatus* NBRC 14919^T^ having the lowest content (63.5%) and *Curvibacter cyanobacteriorum* HBC61^T^ showing the highest content (67.15%). There was a significant difference in the numbers of predicted genes among *Curvibacter* species, which ranged from 3678 to 6367.

### Whole-Genome-Based Phylogeny

**Fig. 2:**
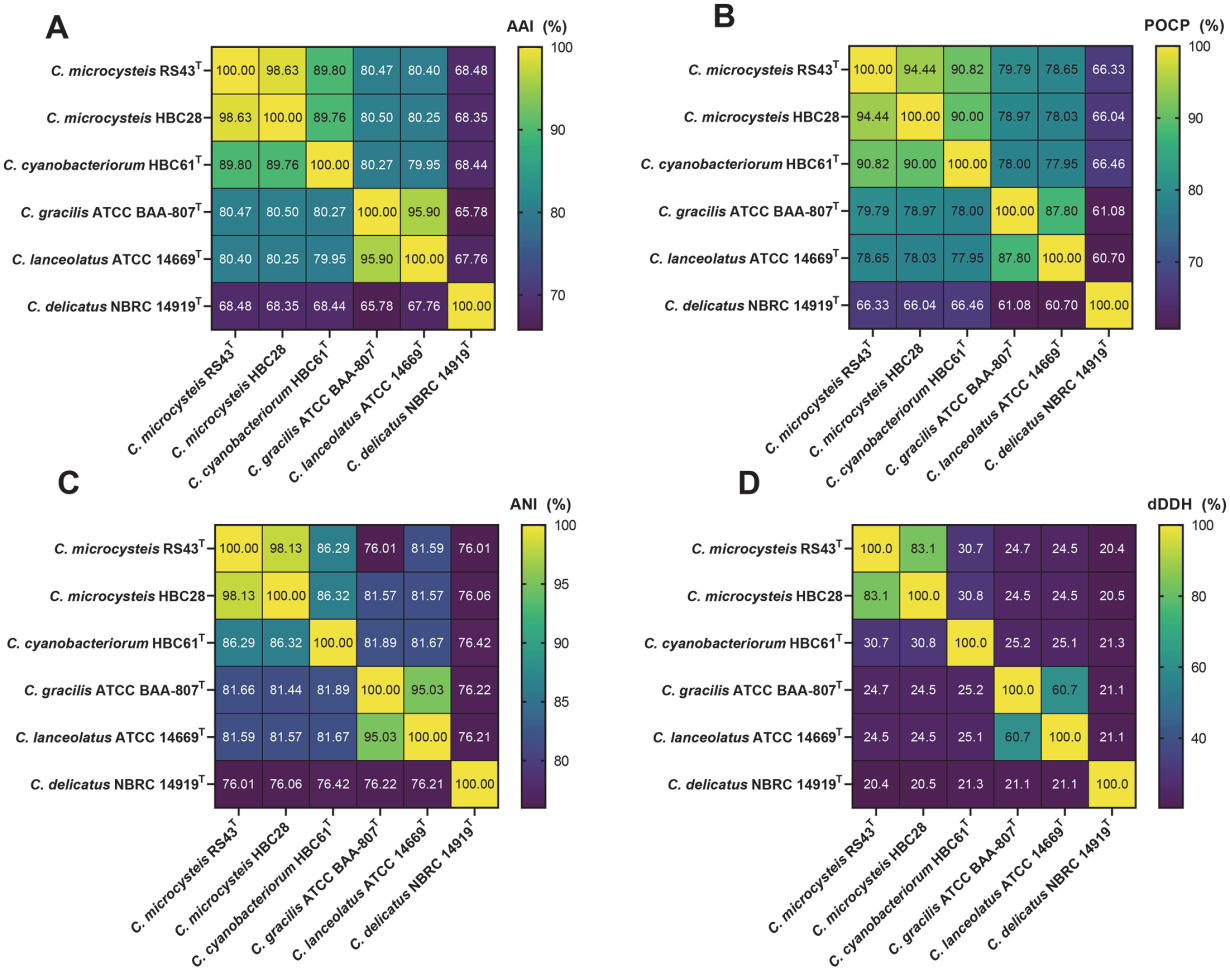
Heat map of AAI (A), POCP (B), ANI (C), and dDDH (D) from pairwise genome comparisons. AAI, average amino acid identity; POCP, percentage of conserved proteins; ANI, average nucleotide identity; dDDH, digital DNA– DNA hybridization.

**Fig. 3:**
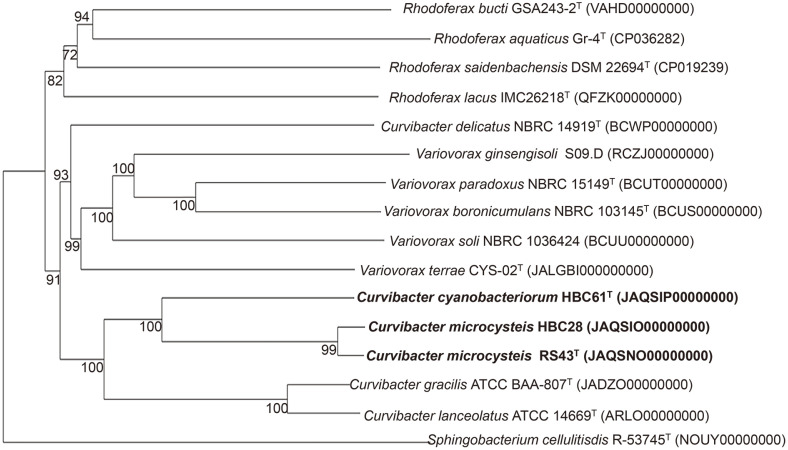
Phylogenomic tree inferred with FastME 2.1.6.1 from Genome BLAST Distance Phylogeny (GBDP) distances calculated from genome sequences. The branch lengths are adjusted with the GBDP distance formula d5. The GBDP pseudo-bootstrap support values from 100 replications exceeding 60% are displayed above the branches. The average support across branches is 94.6%.

The AAI and POCP values for the three strains with the type species of the genus, *C. gracilis*, were found to be between 80.27-80.50% and 78.00-79.79%, respectively, which were higher than the genus boundaries of 60-80%for AAI [31] and 50% for POCP [32] (Figs. 2A-2B). These results supported that the three strains belong to the genus *Curvibacter*. Strains RS43^T^ and HBC28 were found to be the same species with an ANI value of 98.13%(Fig. 2C) and a dDDH value of 83.1% (Fig. 2D). However, the ANI and dDDH values between strains RS43^T^ and HBC61^T^ were 86.29% and 30.7%, respectively, implying that they represent two separate *Curvibacter* species. The dDDH and ANI scores between the three strains and their related type strains were much lower than the recommended species cut-off level (dDDH, 70%; ANI, 95-96%), revealing their novel status within the genus *Curvibacter* [33,34]. The phylogenomic tree showed that the closest neighbors of the three strains were *C. gracilis* 7-1^T^ and *Curvibacter lanceolatus* IAM 14947^T^ (Fig. 3). Notably, *Curvibacter delicatus* formed a distinct clade separated from other members of the genus *Curvibacter*, indicating that this species may belong to other genus. Further studies are needed to identify the exact taxonomic position of this species. In summary, the whole-genome-based phylogeny clearly suggests that strains RS43^T^ and HBC61^T^ should be classified as two novel species within the genus *Curvibacter*.

### Comparative Genomic Analysis

**Fig. 4:**
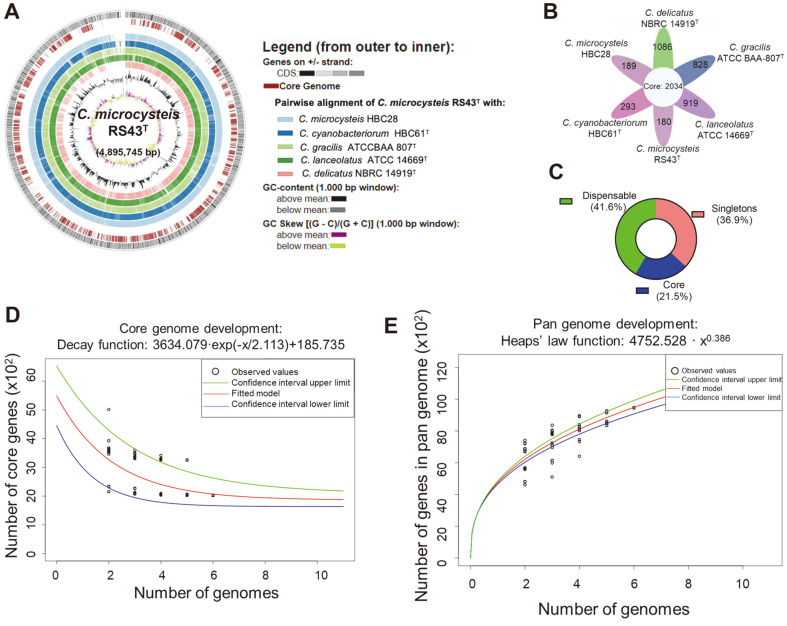
Comparative genomic analysis. Circular view of the genome of *Curvibacter* species (**A**). The number (**B**) and percentage (**C**) of common core genes across all *Curvibacter* species. Core genome (**D**) and pangenome (**E**) profiles of *Curvibacter* species. The fitted exponential Heaps’ law function is represented by the red curve while the upper and lower boundaries of the 95% confidence internal are indicated by the green and blue curves, respectively.

The distribution of specific genomic regions of *Curvibacter* species was shown in Fig. 4A. Functional analysis using the KEGG and COG database revealed that these species displayed similar distribution patterns of subsystem categories (Figs. S1-S2). For instance, most genes were involved in vital central metabolic pathways, such as “amino acid transport and metabolism”, “signal transduction mechanisms”, “transcription”, and “energy production and conversion”. In total, the “core genome” consisted of 2034 genes (Fig. 4B). 41.6% of genes were assigned to the dispensable category, whereas the percentage of singletons was estimated to be 36.9% (Fig. 4C). The lifestyle of the bacterial genus can be partly reflected by its pangenome [35]. Bacteria that inhabit a limited niche tend to have a closed pangenome, whereas bacteria living in a community generally possess an open pangenome with a high rate of horizontal gene transfer [35]. The pangenome is classified as open or closed, based on the exponent gamma value of Heaps’ law [36]. The pangenome size of *Curvibacter* increased while its core-genome size decreased as more genomes were added (Figs. 4D-4E). The gamma value of Heaps’ law was estimated to be 0.386, which is higher than 0, implying that *Curvibacter* pangenome is open (Fig. 4E) [37].

**Fig. 5:**
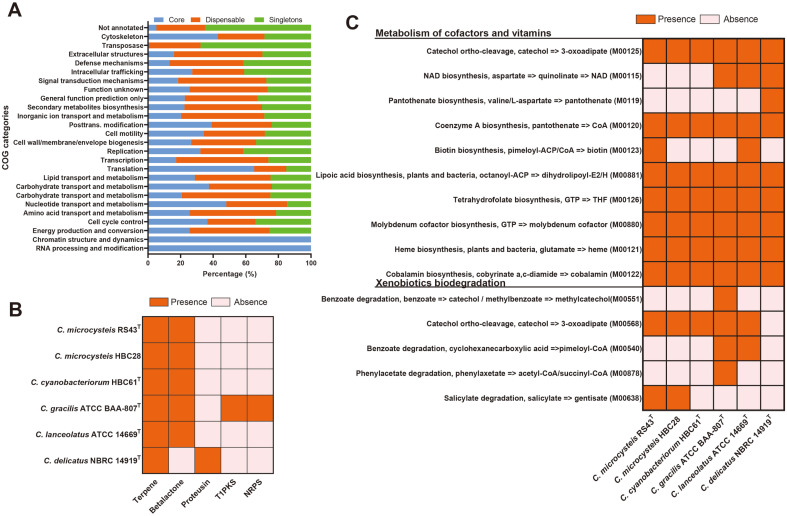
Functional genome analysis of *Curvibacter* species. The distribution of core, dispensable, and singletons (**A**). Gene clusters predicted in the genomes of *Curvibacter* species involved in secondary metabolite synthesis (**B**), Metabolism of cofactors and vitamins and xenobiotics biodegradation in the genomes of *Curvibacter* species (**C**).

The core gene proportion of all strains of *Curvibacter* species was more enriched with COG functions involved in “RNA processing and modification”, “chromatin structure and dynamics”, and “translation”. In contrast, most singleton genes were related to “transposase”, “defense mechanisms”, and “intracellular trafficking” (Fig. 5A). To understand the ecological function of *Curvibacter* species, their potential for vitamin synthesis and biosynthesis of secondary metabolites was predicted. Bacteria produce several secondary metabolites to communicate with other microorganisms [38]. Strains RS43^T^, HBC28, and HBC61^T^ possessed putative secondary metabolite gene clusters potentially related to the synthesis of terpene and beta-lactone (Fig. 5B). Terpene, which was also predicted in all *Curvibacter* species, plays an essential role in the defense mechanisms of plants and fungi [39]. Beta-lactones have been reported to show antimicrobial activities and can be used as building blocks for complex compounds such as antibiotics and anticancer drugs [40]. Among *Curvibacter* species, *C. gracilis* ATCCBAA-807^T^ harbored the highest secondary metabolite biosynthetic gene clusters. This strain can produce terpene, beta-lactone, Type 1 polyketide synthase (T1PKS), and nonribosomal peptide synthetase (NRPS). As NRPS was predicted in the genome of *C. gracilis* ATCCBAA-807^T^, this strain may produce antimicrobial, antiviral, anticancer, and anti-inflammatory compounds [41]. Bacteria can support algal growth by supplying vitamin B and iron [42]. *Curvibacter* species can promote the growth of the cyanobacterium *Microcystis* [43]. Given that *Curvibacter* species possess the biosynthetic pathway for cobalamin (vitamin B12) and biotin (vitamin B7) in their genome (Fig. 5C), they have the potential to provide these vitamins to cyanobacteria, thereby facilitating the formation of cyanobacterial blooms.

Aromatic compounds, which constitute approximately 25% of the world's biomass, are the second most prevalent group of organic compounds in nature after carbohydrates [44]. They are considered one of the most persistent pollutants in the environment [45]. The metabolic capacities of *Curvibacter* species predicted using BlastKOALA suggested that they could metabolize the xenobiotic substances, such as benzoate (*C. gracilis* ATCC BAA-807^T^ and *C. lanceolatus* ATCC 14669^T^), phenylacetate (*C. gracilis* ATCC BAA-807^T^), catechol (all strains except for *C. delicatus* NBRC 14919^T^), and salicylate (RS43^T^ and HBC28) (Fig. 5C). Therefore, they have the potential to remove these compounds from water systems.

### Phenotypic Characteristics

The cells of strains RS43^T^, HBC28, and HBC61^T^ were observed to be Gram-negative, rod-shaped with flagella (Fig. S3), and catalase- and oxidase-positive. The colonies appeared circular, smooth, convex, and colorless with a diameter of 1–2 mm after 3 days of growth on R2A agar medium at 25°C. These strains grew well on R2A and NA media but not on LBA and TSA media. They were positive for hydrolysis of Tween 80, but negative for hydrolysis of Tween 20 and skim milk. Growth occurred within the pH range of 5.5 to 10. Several phenotypic features distinguish strain RS43^T^ and strain HBC61^T^, confirming they belong to two different species. For instance, strain HBC61^T^ assimilated D-mannitol and gluconate, exhibited growth at 40°C, and was able to tolerate NaCl concentration up to 1% (w/v), whereas strain RS43^T^ did not. Table 2 provides further details on the additional phenotypic features distinguishing strains RS43^T^, HBC28, and HBC61^T^ from their closely related strains.

### Chemotaxonomic Characteristics

The fatty acid profile of strains RS43^T^, HBC28, and HBC61^T^ showed a similar pattern to those of the reference strains (Table 3). All strains had major fatty acids (> 10% of total fatty acids) that included summed feature 3 (C_16:1_*ω*7*c* and/or C_16:1_*ω*6*c*), summed feature 8 (C_18:1_ *ω*7*c* and/or C_18:1_ *ω*6*c*), and C_16:0_. Strains RS43^T^, HBC28, and HBC61^T^ possessed Q-8 as the major quinone, which is consistent with the ubiquinone feature of the genus *Curvibacter*. The major polar lipid profile identified in strains RS43^T^ and HBC61^T^ consisted of phosphatidyl-ethanolamine, diphosphatidylglycerol, and unidentified lipids (Fig. S4). However, strain HBC61^T^ could be distinguished from strain RS43^T^ by the presence of an additional unidentified phospholipid as a major component.

### Description of *Curvibacter microcysteis* sp. nov.

*Curvibacter microcysteis* sp. nov. (mi.cro.cys'te.is N.L. gen. n. *microcysteis* of the cyanobacterial genus *Microcystis*). Cells are rod-shaped, 1.2–2.8 μm in length and 0.7–1.0 μm in width. Colonies grown on R2A agar are smooth, convex, and colorless with a 1–2 mm diameter. Motile with peritrichous flagella. Growth was observed at the temperature range of 10–37°C, up to 0.5% NaCl, and pH 5.5–10. Catalase and oxidase are positive. Able to hydrolyze Tween 80. Positive for esterase (C4), esterase lipase (C8), leucine arylamidase, acid phosphatase, and naphthol-AS-BI-phosphohydrolase. The major fatty acids were summed feature 3 (C_16:1_ *ω*7*c* and/or C_16:1_ *ω*6*c*), summed feature 8 (C_18:1_ *ω*7*c* and/or C_18:1_ *ω*6*c*), and C_16:0_. The quinone was Q-8. The major polar lipids were phosphatidylethanolamine, diphosphatidylglycerol, and two unidentified lipids.

The type strain RS43^T^ (=KCTC 92793^T^ = LMG 32714^T^) was isolated from fresh water. The 16S rRNA gene sequence and the genomic sequence of strain RS43^T^ have been deposited under the GenBank/EMBL/DDBJ accession numbers OQ642155 and JAQSIN000000000, respectively.

### Description of *Curvibacter cyanobacteriorum* sp. nov.

*Curvibacter cyanobacteriorum* sp. nov. (cy.a.no.bac.te.ri.órum. N.L. gen. pl. n. *cyanobacteriorum* of cyanobacteria).

Cells are rod-shaped, 1.2–2.7 μm in length and 0.7–0.9 μm in width. Colonies grown on R2A agar are smooth, convex, and colorless with a 1–2 mm diameter. Motile with monotrichous flagellum. Growth was observed at the temperature range of 10–40°C, up to 1% NaCl, and pH 5.5–10. Catalase and oxidase are positive. Able to hydrolyze Tween 80. Positive for esterase (C4), esterase lipase (C8), leucine arylamidase, acid phosphatase, and naphthol-AS-BI-phosphohydrolase, hydrolysis of urea, and assimilation of D-mannitol and potassium gluconate. The major fatty acids were summed feature 3 (C_16:1_ *ω*7*c* and/or C_16:1_ *ω*6*c*), summed feature 8 (C_18:1_ *ω*7*c* and/or C_18:1_ *ω*6*c*), and C_16:0_. The quinone was Q-8. The major polar lipids were phosphatidylethanolamine, diphosphatidylglycerol, one unidentified phospholipid, and two unidentified lipids.

The type strain HBC61^T^ (=KCTC 92794^T^ = LMG 32713^T^) was isolated from fresh water. The 16S rRNA gene sequence and the genomic sequence of strain HBC61^T^ have been deposited under the GenBank/EMBL/DDBJ accession numbers OQ642154 and JAQSIP000000000, respectively.

## Conclusions

Considering chemotaxonomic, phylogenetic, and phenotypic characteristics, strains RS43^T^, HBC28, and HBC61^T^ belonged to the genus *Curvibacter*. Genome-relatedness indices, phenotypic and chemotaxonomic characteristics precisely differentiated them from all known *Curvibacter* species. Along with phylogenetic distinctiveness, strain HBC61^T^ was distinguished from RS43^T^ and HBC28 by several phenotypic and chemotypic features. Therefore, strains RS43^T^, HBC28, and HBC61^T^ should be classified as two novel species of the genus *Curvibacter*, for which the names *Curvibacter microcysteis* sp. nov. (RS43^T^ = KCTC 92793^T^ = LMG 32714^T^) and *Curvibacter cyanobacteriorum* sp. nov. (HBC61^T^ = KCTC 92794^T^ = LMG 32713^T^).

## Supplemental Materials

Supplementary data for this paper are available on-line only at http://jmb.or.kr.



## Figures and Tables

**Table 1:** General genome characteristics of RS43^T^, HBC28, and HBC61^T^ and related taxa.

Species	Strain	Accession No.	Size (Mb)	G+C (%)	Gene	Protein
*Curvibacter microcysteis*	RS43^T^	JAQSIN000000000	4.89	65.22	4,382	4,297
*Curvibacter microcysteis*	HBC28	JAQSIO000000000	4.82	65.29	4,399	4,256
*Curvibacter cyanobacteriorum*	HBC61^T^	JAQSIP000000000	4.85	67.15	4,266	4,185
*Curvibacter gracilis*	ATCC BAA-807^T^	JADZ00000000	6.75	66.01	6,214	6,036
*Curvibacter lanceolatus*	ATCC 14669^T^	ARLO00000000	6.83	65.55	6,367	6,157
*Curvibacter delicatus*	NBRC 14919^T^	BCWP00000000	3.78	63.51	3,678	3,543

**Table 2:** Differential phenotypic characteristics of strains RS43^T^, HBC28, and HBC61^T^ and their phylogenetic neighbors.

Characteristic	1	2	3	4	5	6
Hydrolysis of Tween 20	-	-	-	+	-	-
Optimal growth range						
pH	7.5-8.5	7.5-8.5	6.0-8.0	5.0-8.0^a^	neutrophilic^b^	5.5-8.5^c^
Temperature (°C)	20-30	20-30	20-37	25-30^a^	20-30^b^	30-32^c^
Biochemical characteristics (API ZYM, API 20NE)						
Esterase (C4)	+	+	+	+	+	-
Esterase lipase (C8)	+	+	+	+	+	-
Cystine arylamidase	-	-	-	+	+	-
Reduction of nitrates to nitrites	-	-	-	+	+	+
Urea hydrolysis	-	+	+	+	+	+
Assimilation of						
D-Glucose	-	-	-	+	-	-
D-Mannitol	-	-	+	-	-	-
Gluconate	-	-	+	+	-	-

Taxa: 1, strain RS43^T^; 2, HBC28; 3, HBC61^T^; 4, *Curvibacter gracilis* KCTC42831^T^; 5, *Curvibacter lanceolatus* KCTC42829^T^; 6, *Curvibacter delicatus* KCTC 42828^T^. +, positive; −, negative. ^a^Data were obtained from [3]; ^b^Data were obtained from [4]; ^c^Data were obtained from [46].

**Table 3:** Fatty acid compositions (%) of strains RS43^T^, HBC28, and HBC61^T^ and their phylogenetic neighbors.

Fatty Acids	1	2	3	4	5	6
C_8:0_ 3-OH	4.4	4.2	3.7	3.7	ND	4.2
C_12:0_	4.4	3.7	3.6	3.3	3.7	ND
C_14:0_	ND	ND	ND	ND	TR	ND
C_16:0_	**14.3**	**15.9**	**16.2**	**20.8**	**20.1**	**33.2**
C_17:1_ ω6c	ND	ND	ND	1.44	TR	ND
C_17:0_ cyclo	ND	ND	ND	ND	ND	5.8
C_18:1_ ω9c	ND	ND	TR	TR	ND	1.1
C_18:1_ ω7c 11-methyl	1.03	TR	TR	ND	TR	TR
Summed Feature 3*	**42.8**	**43.3**	**43.1**	**42.6**	**43.1**	**39.0**
Summed Feature 8*	**32.6**	**31.6**	**30.4**	**26.3**	**29.5**	**15.7**

Taxa: 1, strain RS43^T^; 2, HBC28; 3, HBC61^T^; 4, *Curvibacter gracilis* KCTC42831^T^; 5, *Curvibacter lanceolatus* KCTC42829^T^; 6, *Curvibacter delicatus* KCTC 42828^T^. Major fatty acids (> 10.0%) are highlighted as bold type. TR, trace amount (< 1%); ND, not detected. *Summed features refer to fatty acids that cannot be distinctly differentiated from another fatty acid and these fatty acids are grouped as one feature with a single percentage of the total. Summed feature 3 contained C_16:1_ *ω*6*c* and/or C_16:1_ *ω*7*c*. Summed feature 8 contained C_18:1_ *ω*7*c* and/or C_18:1_ *ω*6*c*.
