# Supplementary material for: Comparative Genome analysis of the Genus Curvibacter and the Description of Curvibacter microcysteis sp. nov. and Curvibacter cyanobacteriorum sp. nov., Isolated from Fresh Water during the Cyanobacterial Bloom Period
Source: J Microbiol Biotechnol. 2023 Aug 21;33(11):1428–36. doi: 10.4014/jmb.2306.06017 (PMC10699270; doi:10.4014/jmb.2306.06017)
Supplement: Supplementary file 1 [file jmb-33-11-1428-supple.pdf]

J. Microbiol. Biotechnol.
https://doi.org/10.4014/jmb.2306.06017

# Supplementary Figures

## Genomic characterization of the genus *Curvibacter* and the description of *Curvibacter microcysteis* sp. nov and *Curvibacter cyanobacteriorum* sp. nov., isolated from a eutrophic reservoir

**Ve Van Le[1], So-Ra Ko[1], Mingyeong Kang[1,2], Seonah Jeong[1], Hee-Mock Oh[1,2], and Chi-Yong Ahn[1,2*]**

[1]Cell factory Research Centre, Korea Research Institute of Bioscience & Biotechnology, 125 Gwahak-ro, Yuseong-gu, Daejeon 34141, Republic of Korea

[2]Department of Environmental Biotechnology, KRIBB School of Biotechnology, University of Science and Technology, Daejeon 34113, Republic of Korea

**[*]Corresponding author**

**Chi-Yong Ahn**

**E-mail: cyahn@kribb.re.kr**

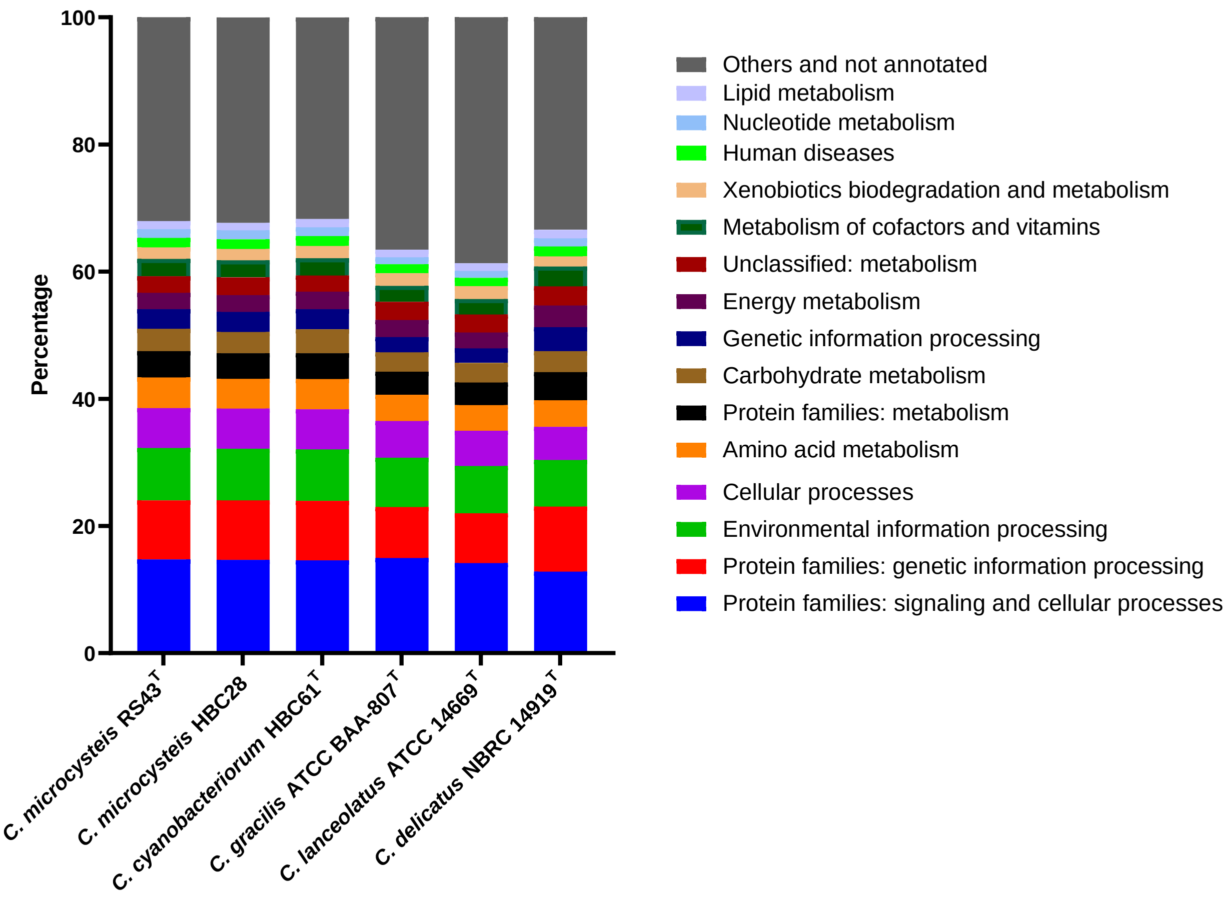


**Fig. S1. Kyoto Encyclopedia of Genes and Genomes (KEGG) analysis of the genome of *Curvibacter* species.**

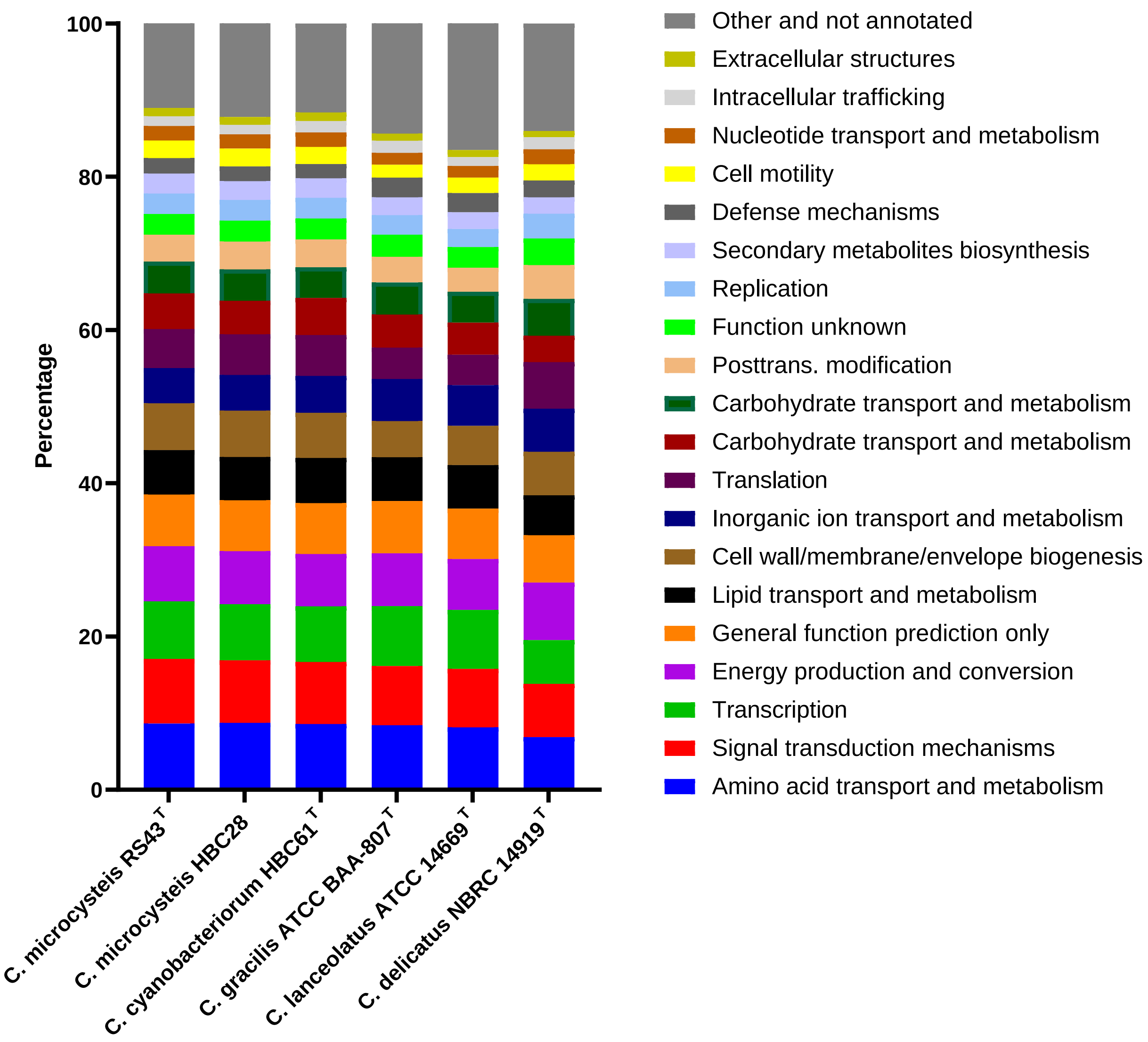


**Fig. S2. Clusters of Orthologous Groups (COG) analysis of the genome of *Curvibacter* species.**

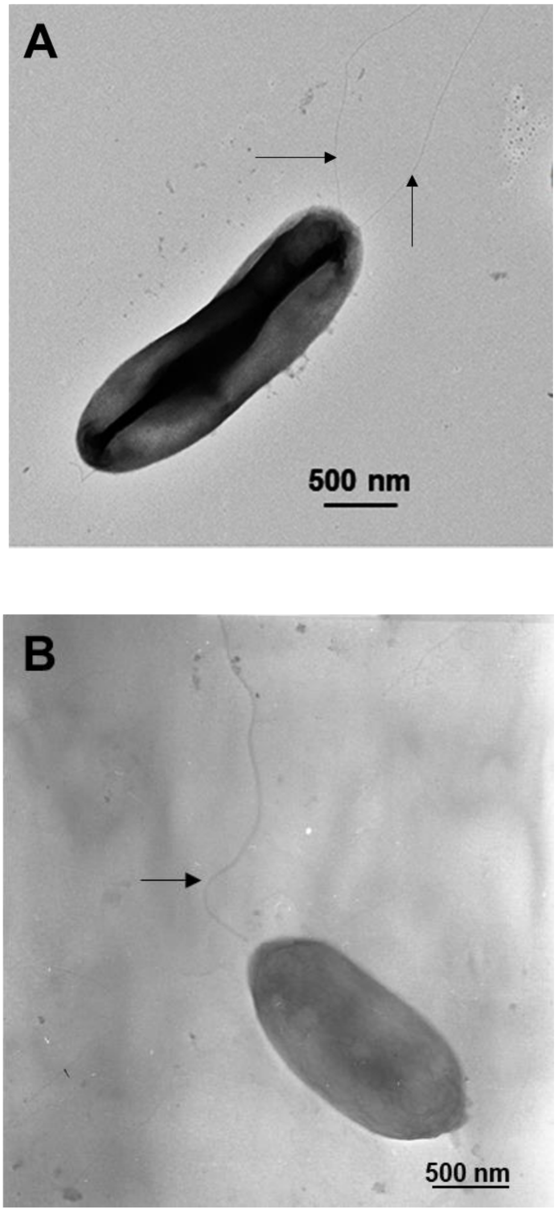


**Fig. S3. Morphology of strain RS43$^{T}$ (A) and HBC61$^{T}$ (B). Transmission electron micrograph**: bar, 500 nm. The cells were grown on R2A at 25°C for 3 days. Arrows indicate flagella.

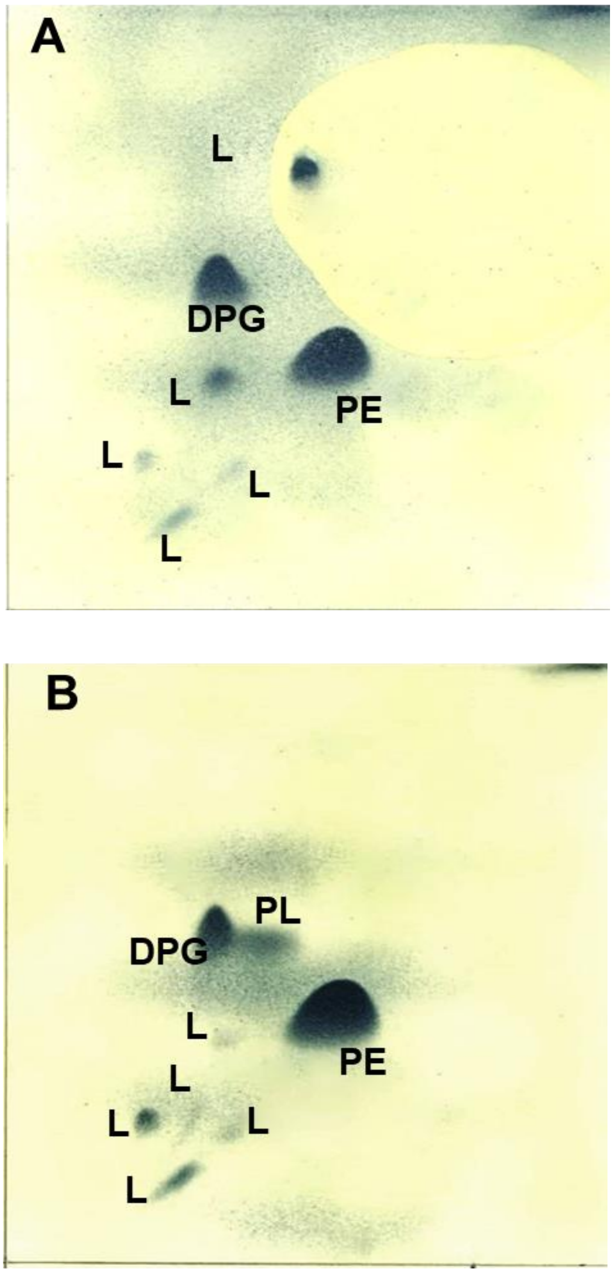


**Fig. S4.** Two-dimensional thin layer chromatography of polar lipids extracted from strain RS43$^{T}$ (A) and HBC61$^{T}$ (B). PE, phosphatidylethanolamine; DPG, diphosphatidylglycerol; PL, unidentified phospholipid; L, unidentified lipid.
